# Associations between the Genetic Polymorphisms of Osteopontin Promoter and Susceptibility to Cancer in Chinese Population: A Meta-Analysis

**DOI:** 10.1371/journal.pone.0135318

**Published:** 2015-08-12

**Authors:** Yulan Liu, Hongbo Lei, Jixiang Zhang, Jun Wang, Kui Li, Weiguo Dong

**Affiliations:** 1 Department of Gastroenterology, Renmin Hospital of Wuhan University, Wuhan, China; 2 Department of Oncology, Renmin Hospital of Wuhan University, Wuhan, China; Shanghai Jiao Tong University School of Medicine, CHINA

## Abstract

**Background and Aim:**

Several studies have been conducted to examine the associations between osteopontin (OPN) promoter gene *SPP1* polymorphisms with human cancers in Chinese population, but the results remain inconsistent. The aim of this meta-analysis is to clarify the associations between *SPP1* polymorphisms and cancer susceptibility.

**Methods:**

All eligible case-control studies published up to March 2015 were identified by searching PubMed, Web of Science, Embase, and Cochrane Library without language restrictions. Pooled odds ratio (OR) and 95% confidence interval (95% CI) were calculated using fixed- or random-effect model.

**Results:**

A total of 11 case-control studies were included; of those, there were eleven studies (3130 cases and 3828 controls) for -443T>C polymorphism, ten studies (3019 cases and 3615 controls) for -156G>GG polymorphism, eight studies (2258 cases and 2846 controls) for -66T>G polymorphism. Overall, no evidence indicated that the -443 T>C polymorphism was associated with cancer risk (OR = 0.93, 95%CI 0.62–1.38 for dominant model, OR = 1.06, 95%CI 0.73–1.55 for recessive model, OR = 0.88, 95%CI 0.62–1.26 for CT vs TT model, OR = 1.03, 95%CI 0.61–1.73 for CC vs TT model). While, a significantly increase risk was found for -156 G>GG polymorphism (OR = 1.22, 95%CI 1.10–1.35 for dominant model, OR = 1.25, 95%CI 1.10–1.41 for recessive model, OR = 1.18, 95%CI 1.06–1.32 for GGG vs GG model, OR = 1.35, 95%CI 1.09–1.68 for GGGG vs GG model). For -66T>G polymorphism, we found a decrease risk of cancer (OR = 0.84, 95% CI 0.71–0.98 for dominant model), but this result changed (OR = 0.93, 95% CI 0.77–1.12 for dominant model) when we excluded a study.

**Conclusion:**

This meta-analysis suggests that in Chinese population the -156G>GG polymorphism of *SPP1* might be a risk factor for human cancers, while -443T>C mutation is not associated with cancer risk. For -66T>G polymorphism, it may be a protective factor for human cancers.

## Introduction

Cancers contribute a greatest deal to death worldwide [1], as a result of interactions between genetic mutation accumulation and environment risk factors. Genetic variation plays an important role in the tumorigenesis with the effect on gene structure and protein expression [2]. Several polymorphisms that have relationship with cancers in the human osteopontin (OPN) encoding gene *SPP1* have been searched.

OPN is a secreted glycophosphoprotein that may physiologically serve as a cytokine and an extracellular matrix molecule. It is expressed and secreted by various cells, and plays a role in bone remodeling, reconfiguration of tissue integrity during inflammatory processes, coronary restenosis, and cancer metastatic [3–6]. It has been demonstrated that OPN is associated with more than 30 cancers so far and a marker for breast, cervical, colorectal, head and neck, liver, lung, ovarian and prostate cancers, as well as for sarcoma [7,8]. The tight correlation with tumor metastatic and progression was initially reported in 1979 [9] and had been approved by many studies, however, the association between OPN and carcinogenesis has just been researched recently [10,11]. The expression of OPN was significantly influenced by its genetic polymorphisms of the promoter [12], *SPP1* (mapped to chromosome 4q24-q25), which is predominantly a transcriptionally regulated gene with highly conserved promoter [13]. Several polymorphisms in the *SPP1* gene affect OPN expression and the level of its secretion into bovine milk [14]. Common single nucleotide polymorphisms (SNPs) such as -443C>T (rs11730582), -156G>GG (rs17524488) and -66T>G (rs28357094) may result in increased expression of *SPP1* gene and tumor risk. The results remain inconclusive and a comprehensive analysis is necessary. Therefore, we implemented a meta-analysis that integrated all studies for *SPP1*polymorphisms and risk for all types of human cancer in order to obtain an accurate assessment.

## Materials and Methods

### Search strategy

A literature research was conducted using PubMed, Web of Science, Embase, and Cochrane Library up to March 2015 without language restrictions. Relevant studies were searched using the terms [osteopontin or OPN or *SPP1*] AND [-443C>T or rs11730582 or -156G>GG or rs17524488 or -66T>G or rs28357094] AND [variant or genetic polymorphism or polymorphism or mutation]. Additional studies were identified by screening references in the retrieved articles and preceding reviews on the topic.

### Inclusion criteria and exclusion criteria

Studies were included if they met the following criteria: (1) case–control study; (2) about the associations between *SPP1* polymorphisms (-443T>C or -156G>GG or -66T>G) and cancer risks; and (3) had available genotype frequencies of cases and controls or could be calculated from the paper. Accordingly, the exclusion criteria were (1) duplicate data, (2) only for cancer samples, (3) only for benign disease compared with controls, and (4) number of the cases less than 30.

### Data extraction and quality assessment

Two of the authors independently selected the article and extracted data with consensus on all of the terms. If the data was not identical, the two investigators would check the data again to come to an agreement. If they could not reach an agreement, an expert (Weiguo Dong) would participate in the discussion. Following items were collected from the eligible articles: first author’s name, year of publication, country of origin, ethnicity, cancer type, number of cases and controls, age, gender, OPN levels and genotypes distributions in cases and controls.

The quality of selected studies was independently evaluated on basis of Newcastle-Ottawa scale (NOS) [15]. Studies with six or more stars were considered as high quality.

### Statistical analysis

Meta-analysis was performed using the Cochrane Collaboration Revman 5.3 (Copenhagen, 2014) and STATS package version 9.2 (Stata corporation, College Station, Texas). The risk of cancer associated with three polymorphisms respectively of *SPP1* gene was estimated for each eligible study by odds ratio (OR) and 95% confidence interval (95% CI). We used χ^2^-based Q statistic text [16] and *I* ^*2*^ index [17] to assess the heterogeneity between the studies. When heterogeneity across studies (Q test P≤0.05 or *I* ^*2*^ >50%) was showed, random-effect model was used [18], otherwise, the fixed-effects model was used [19]. Hardy-Weinberg equilibrium (HWE) in control people was judged by χ^2^ text. We evaluated the associations of three polymorphisms with cancer risk under dominant, recessive, codominant, and heterozygote models respectively. Then, we analyzed the sensitivity to evaluate the stability of results after removing the studies deviating from HWE. Publication bias was diagnosed with Begg’s funnel plot [20] and Egger’s linear regression [21]. P<0.05 was regarded as a state of disequilibrium.

## Results

### Study characteristics

**Fig 1:**
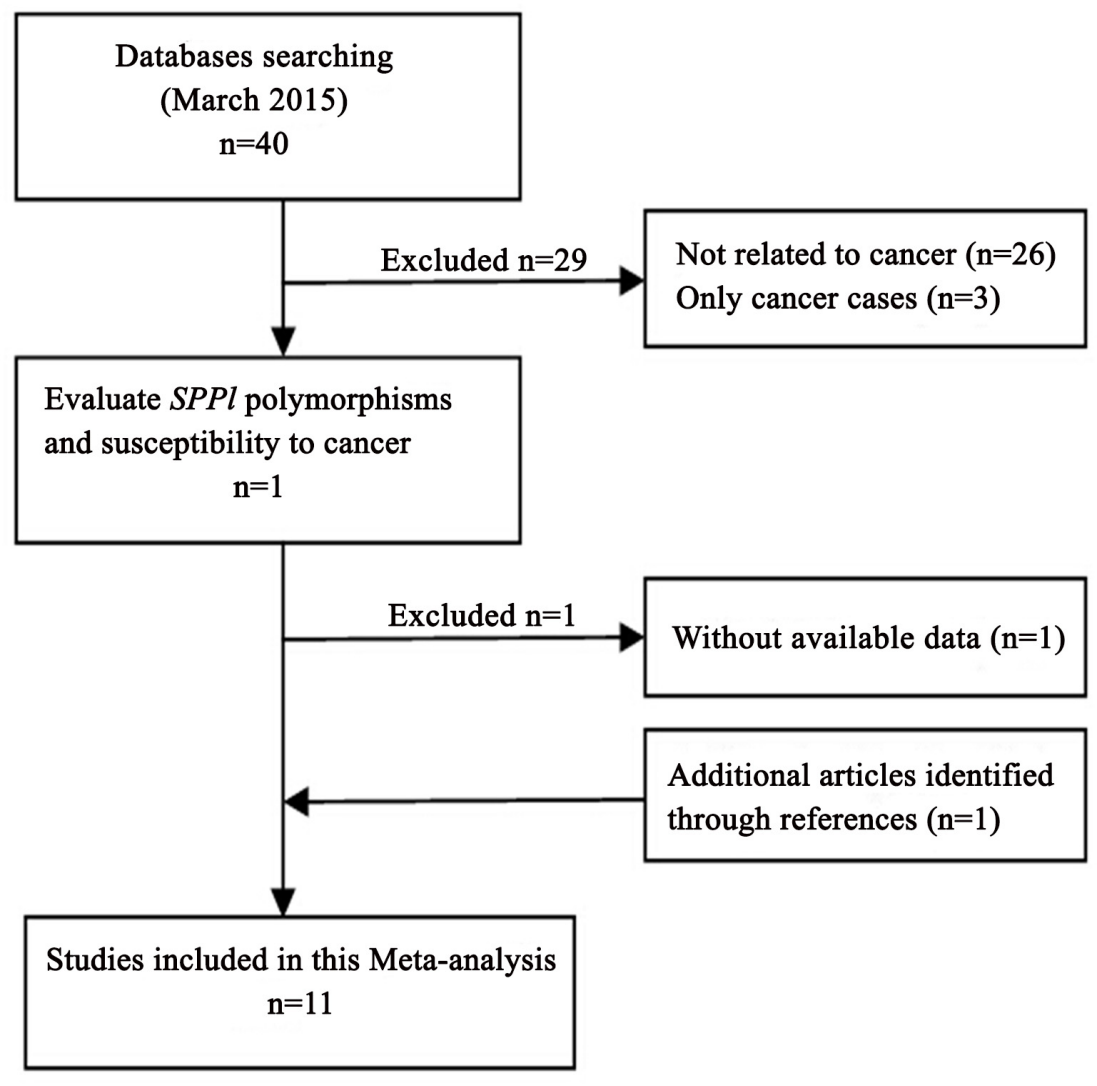
Flow chart showing study selection procedure.

The search strategy retrieved 40 potential relevant studies and one study was identified through references. According to the inclusion and exclusion criteria, 11 studies [10,12,22–30]with full text were eligible for this meta-analysis and 30 studies were excluded. The flow chart of study selection is summarized in Fig 1. All studies taken in China and all participators came from Chinese population. There were eleven case-control studies with 3130 cancer cases and 3828 controls concerning -443T>C polymorphism, ten case-control studies with 3019 cases and 3615 controls concerning -156 G>GG, and eight case-control studies with 2258 cases and 2846 controls concerning -66T>G. Cancer types include glioma, non-small-cell lung cancer (NSCLC), oral squamous cell carcinoma (OSCC), gastric cancer (GC), papillary thyroid cancer (PTC), nasopharyngeal carcinoma (NPC), cervical cancer, acute myeloid leukemia (AML), and intrahepatic cholangiocarcinoma (ICC). Blood samples were used to determine genetic polymorphisms in all of the included studies. The distribution of genotypes in the controls was consistent with HWE for all selected studies except for two studies [23,29]. The qualities of all included studies were categorized as high quality. Table 1 showed the characteristics and NOS quality of the enrolled studies.

**Table 1:** Characteristics of studies included in the meta-analysis.

Study	Year	Cancer type	Number	Age, mean ± SD, year		Gender (male, %)	Genotype (case/control)			P_HWE_	NOS
			Case/Control	Case	Control	Case/Control	WT Ho	Ht	VR Ho		
-443T>C											
Chen JX	2010	Glioma	670 /680	NR	NR	59.7/54.6	299/284	299/311	69/77	0.557	7
Chen YZ	2013	NSCLC	360/360	57.2	56.3	55.3/54.7	164/153	165/163	31/44	0.954	7
Chiu YW	2010	OSCC	97/100	53.6	NR	88.7/NR	47/33	41/50	9/17	0.793	8
Lee TY	2013	GC	146/128	64.0±13.9	61.4±8.5	59.6/57.0	59/65	66/55	21/8	0.416	7
Mu GY	2013	PTC	363/413	38.6±2.1	38.4±4.3	39.4/49.4	73/164	171/187	119/62	0.469	8
Shen ZP	2014	Glioma	248/281	45.2±3.5	44.9±4.1	46.7/45.7	54/90	113/137	81/54	0.885	9
Wang JL	2014	NPC	108/210	48.2±10.5	47.8±11.2	66.7/57.1	60/85	38/95	10/30	0.678	8
Xu Q	2011	Cervical cancer	300/774	54.6±5.7	54.5±2.6	0/0	227/343	49/334	24/106	0.126	7
Zhang R	2015	AML	381/430	54.5±3.6	54.6±4.1	51.7/52.1	81/117	183/223	117/90	0.392	7
Zhao FJ	2012	GC	200/200	56.3±3.5	55.7±4.2	65.0/65.0	91/85	94/93	15/22	0.646	7
Zhao XQ	2014	ICC	260/260	57.2±NR	56.3±NR	57.3/56.5	120/114	111/115	29/31	0.809	8
-156G>GG											
Chen JX	2010	Glioma	670 /680	NR	NR	59.7/54.6	220/273	345/306	99/90	0.772	7
Chen YZ	2013	NSCLC	360/360	57.2	56.3	55.3/54.7	137/155	150/136	73/69	0.0001	7
Chiu YW	2010	OSCC	97/100	53.6	NR	88.7/NR	27/42	52/49	18/9	0.318	8
Lee TY	2013	GC	146/128	64.0±13.9	61.4±8.5	59.6/57.0	48/46	72/64	26/18	0.911	7
Mu GY	2013	PTC	363/413	38.6±2.1	38.4±4.3	39.4/49.4	104/100	187/219	72/94	0.217	8
Shen ZP	2014	Glioma	248/281	45.2±3.5	44.9±4.1	46.7/45.7	57/67	124/153	67/61	0.134	9
Xu Q	2011	Cervical cancer	300/774	54.6±5.7	54.5±2.6	0/0	88/287	129/359	83/128	0.318	7
Zhang R	2015	AML	381/430	54.5±3.6	54.6±4.1	51.7/52.1	84/114	198/226	99/90	0.259	7
Zhao FJ	2012	GC	200/200	56.3±3.5	55.7±4.2	65.0/65.0	67/86	92/78	41/36	0.017	7
Zhao XQ	2014	ICC	260/260	57.2±NR	56.3±NR	57.3/56.5	111/107	101/110	48/43	0.109	8
-66T>G											
Chen YZ	2013	NSCLC	360/360	57.2	56.3	55.3/54.7	356/351	4/9	0/0	0.81	7
Lee TY	2013	GC	146/128	64.0±13.9	61.4±8.5	59.6/57.0	146/128	0/0	0/0	NA	7
Mu GY	2013	PTC	363/413	38.6±2.1	38.4±4.3	39.4/49.4	99/114	167/191	97/108	0.128	8
Shen ZP	2014	Glioma	248/281	45.2±3.5	44.9±4.1	46.7/45.7	83/88	130/147	35/46	0.239	9
Xu Q	2011	Cervical cancer	300/774	54.6±5.7	54.5±2.6	0/0	97/181	199/210	93/121	0.668	7
Zhang R	2015	AML	381/430	54.5±3.6	54.6±4.1	51.7/52.1	89/99	199/210	93/121	0.668	7
Zhao FJ	2012	GC	200/200	56.3±3.5	55.7±4.2	65.0/65.0	200/200	0/0	0/0	NA	7
Zhao XQ	2014	ICC	260/260	57.2±NR	56.3±NR	57.3/56.5	256/251	4/9	0/0	0.776	8

P_HWE_ was calculated by goodness-of fit χ^2^-test, P_HWE_ <0.05 was considered statistically significant; Ht, heterozygote; HWE, Hardy–Weinberg equilibrium; NA, not available; VR Ho, variant homozygote; WT Ho, wild-type homozygote.

### Quantitative data synthesis

For -443T>C polymorphism, eleven case-control studies [10,12,22–30] with 3130 cases and 3828 controls were identified. Overall, there is no significant difference in -443T>C genotype distribution between cancer and control [dominant model (OR = 0.93, 95%CI 0.62–1.38, *P*<0.0001); recessive model (OR = 1.06, 95%CI 0.73–1.55, *P*<0.0001); CT vs TT model (OR = 0.88, 95%CI 0.62–1.26, *P*<0.0001); CC vs TT model (OR = 1.03, 95%CI 0.61–1.73, *P*<0.0001)] (Table 2).

**Table 2:** Summary of ORs of the *SPP1* polymorphisms and cancer risk.

SNP	n	Dominant model			Recessive model			Ht versus WT Ho			VR Ho versus WT Ho		
		OR(95% CI)	P^a^	I^2^	OR(95% CI)	P	I^2^	OR(95% CI)	P	I^2^	OR(95% CI)	P	I^2^
-443T/C	11	0.93(0.62,1.38)	.000	93%	1.06(0.73,1.55)	.000	85%	0.88(0.62,1.26)	.000	90%	1.03(0.61,1.73)	.000	91%
-156G/GG													
Total	10	1.22(1.10,1.35)	.09	40%^b^	1.25(1.10,1.41)	.07	43%^b^	1.18(1.06,1.32)	.15	32%^b^	1.35(1.09,1.68)	.03	52%
Studies with HWE	8	1.20(1.07,1.35)	0.05	50%^b^	1.29(1.05,1.60)	.07	53%	1.13(1.00,1.28)	0.16	33%^b^	1.37(1.05,1.80)	0.01	62%
-66T/G	8	0.84(0.71,0.98)	.15	38%^b^	1.02(0.79,1.32)	.07	57%	0.76(0.54,1.08)	.006	70%	0.91(0.74,1.10)	.87^b^	0

n: number of studies.

^a^Test for heterogeneity.

^b^Fixed-effect model was used when the P for heterogeneity test was>0.05 or I^2^≤50%, otherwise the random-effect model was used.

CI, confidence interval; OR, odds ratio; SNP, single-nucleotide polymorphism; Ht+VR Ho vs WT Ho, dominant model; VR Ho vs Ht+WT Ho, recessive model.

For -156G>GG polymorphism, ten case-control studies [12,22–30] with 3019 cases and 3615 controls were identified. Overall, a significant increased risk was found under all four models [dominant model (OR = 1.22, 95%CI 1.10–1.35, *P* = 0.09); recessive model (OR = 1.25, 95%CI 1.10–1.41, *P* = 0.07); GGG vs GG model (OR = 1.18, 95%CI 1.06–1.32, *P* = 0.15); GGGG vs GG model (OR = 1.35, 95%CI 1.09–1.68, *P* = 0.03)] (Table 2). We evaluated the influence of these studies on the pooled OR by deleting the studies that were not in HWE from the meta-analysis. The estimated pooled odd ratio still did not change at all (Table 2).

For -66T>G polymorphism, eight case-control studies [23–30] with 2258 cases and 2846 controls were identified. Overall, we found that a significant decreased risk under dominant model (OR = 0.84, 95%CI 0.71–0.98, *P* = 0.15), but no significant association was found under other three models (Table 2).

### Heterogeneity and sensitivity analysis

**Fig 2:**
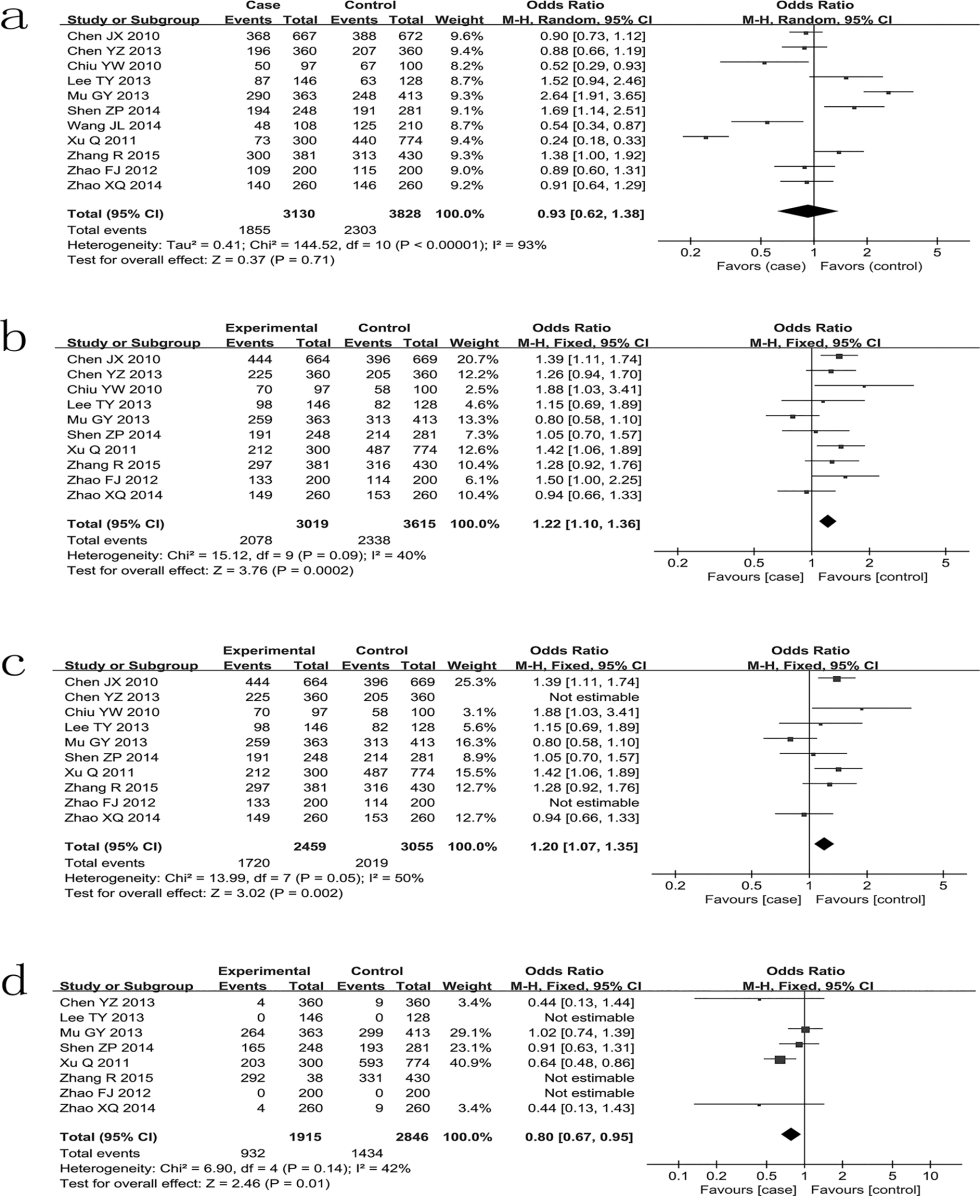
Meta-analysis of the association between *SPP1* polymorphisms and susceptibility to cancer under dominant model. (a) -443 T>C. (b) -156G>GG. (c)-156G>GG with HWE. (d) -66T>G.

For -443T>C polymorphism, there was significant heterogeneity for overall comparisons under all four models (*P*<0.0001). For -156G>GG polymorphism, significant heterogeneity between studies was observed in overall comparisons under GG/GG versus GG model (*I* ^2^ = 52%, *P* = 0.03). And for -66T>G polymorphism, significant heterogeneity between studies was found in overall comparisons under recessive model and TG versus TT model (*I* ^*2*^ = 57%, *P* = 0.07; *I* ^*2*^ = 0.006, *P* = 70%, respectively). (Fig 2). Then, sensitivity analysis was performed to evaluate the stability of the results by removing one study one by one. For -156G>GG polymorphism, the heterogeneity decreased when exclude the study by Mu GY, so it suggests that Mu GY may be the source of heterogeneity. For -66 T>G polymorphism, the heterogeneity decreased to zero when the study of Xu Q were excluded, so the particular study may be the source of heterogeneity.

### Publication bias

**Fig 3:**
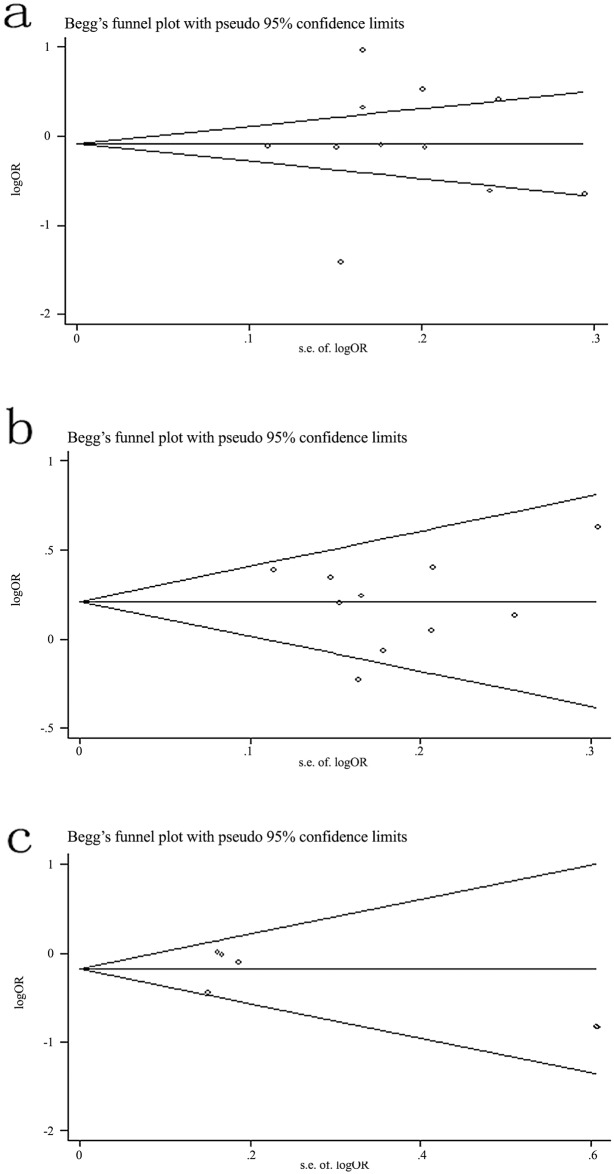
Begg’s funnel plot for publication bias under dominant model. (a) -443T>G. (b) -156G>GG. (c) −66T>G

Begg’s funnel plot and Egger’s test were performed to address potential publication bias in the available literature. The shape of funnel plots did not indicate any evidence of funnel plot asymmetry (Fig 3). Egger’s test also reveal that there was no statistical significance for evaluation of publication bias under dominant model (-443T>C: *P* = 0.818, -156G>GG: *P* = 0.418, -66T>G: *P* = 0.842).

## Discussion

Osteopontin is a member of small intrgrin-binding ligand N-linked glycoproteins (SIBLINGs) family. There are many pieces of evidences indicating that osteopontin profoundly regulate the development and progression of various tumors. Additionally, OPN expression was significantly higher in human cancers tissues than in matched normal tissues and it was significantly associated with nodal metastasis and more advanced clinical stage. Therefore, numerous publications have tested OPN as a biomarker for cancer invasiveness [31–34]. However, the associations with tumorigenesis have not been proved well. Polymorphisms in the OPN gene, *SPP1*, may potentially alter the expression of OPN and then modulate the risk for cancer. In recent years, SNPs have been identified as a powerful tool for predicting some complex diseases. However, previous genetic epidemiological studies about the associations between OPN gene polymorphisms and the risk of human cancer are limited, and the results were inconclusive. To our knowledge, this is the first meta-analysis which investigated the possible correlations of rs11730582 (-443 T>C), rs17524488 (-156G>GG), and rs28357094 (-66T>G) polymorphisms in the *SPP1* gene with cancer susceptibility.

Our results revealed that -443T>C polymorphism might have no relation with pathogenesis of cancer. And we found that different studies had inconsistent results about this polymorphism even for the same cancer. For example, Chen JX [22] said that in a recessive genetic model TC + CC genotypes significantly decrease the risk of glioma when compared with TT, but Shen ZP [26] considered that the glioma patients had markedly high frequency of -443CC genotype than controls. Another two studies [29,30] showed that there was no significant difference in the distribution of -443 between cancer patients and controls. What’s more, large heterogeneity was found in four gene models (P<0.0001). Previous meta-analysis about human cancer risk found that the cancer type might contribute most to the source of heterogeneity [35,36]. In this study, the subgroup analysis on basis of cancer types was not calculated because of the limited number of studies. So this result should be interpreted with caution.

For -156G>GG, our study found that GG allele was at significant high risk for cancer under all four genetic models, and this result was confirmed among studies in HWE. When we exclude the study of Mu GY [25] which may be source of the heterogeneity, the results remain unchanged. That means the SNP of -156G>GG may considerably act a potential candidate of biomarker for cancer risk.

The meta-analysis of -66T>G include eight studies, however, two studies [24,29] that was not estimable in meta-analysis (Fig 2). The results of polymorphism in a dominant model showed that the genotypes TG+ GG significantly decreased the risk of cancer when compared with TT. Noteworthy, the association was disappear when exclude the study of Xu Q [27] that may be the source of the heterogeneity. So, the result is instable and further studies are necessary to clarify the association.

High OPN expression in the primary tumors is associated with cancer risk, metastasis and poor clinical outcome [37–39]. The previous study showed that -443 promoter region exerts influence on OPN gene expression in melanoma cells [40]. In our including studies, four studies [10,25,26,28] observed the association of OPN levels and *SPP1* polymorphisms. Mu GY [25] and Zhang R [28] observed that the high OPN expression was more frequent in samples from -443 CC carriers than TT carriers, However, Shen ZP [26] found that none of the polymorphisms affected the serum OPN levels, Wang JL [10] thought that carriers of CC and CT genotype of -443 presented lower serum osteopontin levels than those of TT genotype. Among the four studies only two gave the accurate data, so we cannot offer further statistics. The result may be caused by following reasons: (1) the *SPP1* polymorphisms affected the tumor OPN expression level, but not the serum OPN level; (2) the association is indeed related and further studies are just needed; (3) *SPP1* polymorphisms make no difference in OPN level.

Some advantage could be highlighted in this meta-analysis. On one hand, this research shed lights on the relationship of genetic polymorphisms in *SPP1* gene and the increased susceptibility to human cancers in Chinese population systematically. One the other hand, the exhaustive inclusion criteria and articles on wide range of cancers enhanced the power and persuasion of our conclusion. Furthermore, all literatures included had acceptable quality scores (scored at least 6). Meanwhile, we were also aware of several limitations of our study. First, all eligible studies come from China and the patients are Chinese population. Second, the number of the studies, especially for -66T>G polymorphism, was not sufficiently large, Third, for -443T>C polymorphism, the heterogeneity was big, the comprehensive analysis should be explain with caution.

## Conclusions

This meta-analysis indicated that in Chinese population the -156G>GG polymorphism of *SPP1* may increase the susceptibility of human cancers, while -443T>C mutation is not associated with cancer risk. For -66T>G polymorphism, it may be a protective factor for human cancers. Accordingly, large and well-designed studies are warranted to validate our findings. The populations in this study only came from China. Thus, populations of other ethnicities should be involved in future studies.

## Supporting Information

S1 Checklist Meta-analysis on Genetic Association Studies checklist. (DOCX)

S1 Checklist PRISMA Checklist. (DOC)
